# Discovery of a Novel Dual Targeting Peptide for Human Glioma: From *In Silico* Simulation to Acting as Targeting Ligand

**DOI:** 10.34172/apb.2024.033

**Published:** 2024-03-10

**Authors:** Negar Sedghi Aminabad, Yousef Saeedi, Jamal Adiban, Mahdieh Nemati, Donya Shaterabadi, Farhood Najafi, Reza Rahbarghazi, Mehdi Talebi, Amir Zarebkohan

**Affiliations:** ^1^Department of Medical Nanotechnology, Advanced Faculty of Medical Sciences, Tabriz University of Medical Sciences, Tabriz, Iran.; ^2^Student Research Committee, Tabriz University of Medical Sciences, Tabriz, Iran.; ^3^Department of Faculty of Life Sciences and Biotechnology, Shahid Beheshti University, Tehran, Iran.; ^4^Ministry of Health and Medical Education, Tehran, Iran.; ^5^Department of Resin and Additives, Institute for Color Science and Technology, Tehran, Iran.; ^6^Stem Cell Research Center, Tabriz University of Medical Sciences, Tabriz, Iran.; ^7^Department of Applied Cell Sciences, Advanced Faculty of Medical Sciences, Tabriz University of Medical, Tabriz, Iran.; ^8^Hematology and Oncology Research Center, Tabriz University of Medical Sciences, Tabriz, Iran.; ^9^Drug Applied Research Center, Tabriz University of Medical Sciences, Tabriz, Iran.

**Keywords:** Leptin-derived peptide, MD simulation, Receptor-mediated transcytosis, Glioma, Brain targeting, PAMAM dendrimer, In vivo

## Abstract

**Purpose:**

Receptor-mediated transcytosis (RMT) is a more specific, highly efficient, and reliable approach to crossing the blood-brain-barrier (BBB) and releasing the therapeutic cargos into the brain parenchyma.

**Methods:**

Here, we introduced and characterized a human/mouse-specific novel leptin-derived peptide using *in silico*, *in vitro* and *in vivo* experiments.

**Results:**

Based on the bioinformatics analysis and molecular dynamics (MD) simulation, a 14 amino acid peptide sequence (LDP 14) was introduced and its interaction with leptin-receptor (ObR) was analyzed in comparison with an well known leptin-derived peptide, Lep 30. MD simulation data revealed a significant stable interaction between ligand binding domains (LBD) of ObR with LDP 14. Analyses demonstrated suitable cellular uptake of LDP 14 alone and its derivatives (LDP 14-modified G4 PAMAM dendrimer and LDP 14-modified G4 PAMAM/pEGFP-N1 plasmid complexes) via ObR, energy and species dependent manner (preferred uptake by human/mouse cell lines compared to rat cell line). Importantly, our findings illustrated that the entry of LDP 14-modified dendrimers in hBCEC-D3 cells not only is not affected by protein corona (PC) formation, as the main reason for diminishing the cellular uptake, but also PC *per se* can enhance uptake rate. Finally, fluorescein labeled LDP 14-modified G4 PAMAM dendrimers efficiently accumulated in the mice brain with lower biodistribution in other organs, in our *in vivo* study.

**Conclusion:**

LDP 14 introduced as a novel and highly efficient ligand, which can be used for drugs/genes delivery to brain tissue in different central nervous system (CNS) disorders.

## Introduction

 During the last decades, the prevalence of brain-related somatic or mental diseases has become one of the challenging issues in clinical setting with socioeconomic outcomes. As a correlate, the delivery of therapeutic compounds to the brain parenchyma turned into the top priority of pharmaceutical nanomedicine.^1-4^ The existence of an impermeable physico/metabolic barrier, namely blood-brain-barrier (BBB), limits the facile entry and access of several therapeutic agents to the neuronal system. In terms of anatomical microstructure, the juxtaposition of multiple cell types such as luminal brain capillary endothelial cells (BCECs) with pericytes, astrocytes, and microglia cells at the abluminal surface makes the BBB a selective cell barrier between the blood and CNS.^5-7^ It has been indicated that compounds with a molecular weight of less than 500 Da and hydrophobic factors can cross the BBB interface.^8,9^ From a physiological aspect, the interactive BBB cell layer is crucial for the function and normal activity of neurons. Following the occurrence of several pathologies and disorders, the BBB interface is the main obstacle to the delivery of therapeutic agents and biological macromolecules like antibodies (Ab), peptides, proteins, peptides, etc. into the brain parenchyma.^10^ Based on several pieces of scientific documents, the development and application of receptor-mediated transcytosis (RMT)-based delivery approaches are efficient in circumventing the limitations associated with the barrier function of BBB. In this regard, the stimulation of target receptors on the luminal side of BCECs with the minimum interaction by other intracellular organelles is done in an energy-dependent manner.^11,12^ For successful interaction, targeting moieties in the structure of ligands and receptors seems inevitable.^9,13^ Of note, the target receptors should be specifically expressed on the surface of BCECs to reduce off-targeting effects. The selection of ligands is based on specific physicochemical and biological properties such as biocompatibility, lack of immunogenicity, specificity, easy and low-cost synthesis steps, suitable size, and shape.^14^ Up to now, several biological ligands derived from hormones, transcriptional factors, and animal venoms including angiopep-2,^15-17^ melittin,^18^ chlorotoxin (Chx),^19-21^ candoxin-derived peptide (CDX),^22,23^ transferrin (Tf),^24-26^ insulin-like growth factor (IGF),^27^ leptin receptor (ObR),^28,29^ etc. have been used for different delivery purposes. Also, enhancing the targeting ability or biocompatibility of peptide ligands by deleting the defects of the structure, is one of the most reliable ways. D8 and Angiopep-2 are two fascinating examples that are in clinical trials for delivery purposes.^30,31^

 Leptin, a 167 amino acid peptide sequence, is an adipose tissue-derived hormone with an average molecular weight of 16kD and several biological properties.^32^ This hormone can modulate the activity of immune cells, the autonomic nervous system, the metabolism rate, and the endocrine system.^33^ Emerging data have revealed the impact of leptin on BBB function.^34^ Crystallographic analysis of leptin has exhibited a long-chain helical structure with the potential to cross the BBB interface following attachment to EC receptors.^35^ Based on several molecular investigations, ObR is expressed in the luminal surface of BBB, blood-CSF barrier, and choroid plexus to uptake leptin and regulate food ingestion and metabolism.^36^ Upon the activation of ObR, leptin enters the brain parenchyma via the activation of transcytosis.^37^ In terms of intracellular domains, six types of ObR have been indicated with different effects on BBB. It has been indicated that OBRa and ObRc are short ObR types with specific activity involved in transcytosis.^38^ Other isoforms, especially type b ObR (ObRb) can regulate cell viability via the alteration of JAK/STAT, mTOR, and PI3K.^39^ In several experiments, numerous leptin-derived peptides have been used for on-target drug delivery to the brain. Data confirmed that Lep 30 is the most efficient leptin derivate for delivery approaches.^28,29^

 Herein, a novel leptin-derived peptide, namely LDP 14, was introduced with suitable targeting efficiency in free and conjugated nanoparticle (NP) forms based on MD simulation, subcellular system and *in vivo* analyses. Noteworthy, LDP 14 possesses functional sequences of all the leptin hormone-derived peptides which can be used for targeting purposes. Also, the possible interfering role of PC formation, as one of the most imperative parameters on the fate of ligand-modified NPs, was evaluated on LDP 14-modified NPs targeting efficiency.

## Material and Methods

###  Materials

 Plasmid pEGFP-N1 (Clontech, Palo Alto, CA, USA) was purified using QIAGEN Plasmid Mega Kit (Qiagen GmbH, Hildden, Germany). LDP 14, Lep 30, SRLC (cyclic), SRLL (linear), and TGN peptides with sequences (ANDLENLRDLLHLLC), (YQQVLTSLPSQNVLQIANDLENLRDLLHLLC), (CLSSRLDAC), (LSSRLDAC), and (TGNYKALHPHNG) were purchased from Biomatic Companies (Wilmington, USA) and Genecust (Boynes, France) with a purity percentage of 95%. Hoechst 33342 and Fluorescein dyes (purity 99%) were provided from Molecular Probes (Eugene, OR, USA). PAMAM G4 dendrimer was synthesized by a two-step iterative process for constructing poly(amidoamine) (PAMAM) dendrimers possessing terminal amine groups (Esfand and Tomalia, 2002). Human super-active leptin antagonist (SHLA, purity 99%) was provided by MyBioSource, Inc. (San Diego, USA). Bifunctional PEG (NHS-PEG-MAL, MW 2000, purity 99%) was purchased from JenKem (Plano TX, USA). Fluorescein fluorophore was purchased from Molecular Probes (Eugene, OR, USA).

###  Cell lines and culture protocols

 C6 rat glioma cells and human U87 glioma cells were purchased from the Iranian National Cell Bank (Pasteur Institute, Tehran). Human brain capillary endothelial cells (hBCEC/D3) were received as a gift from Dr. Emel Sukullu (Koç University, Turkey). Cells were cultured in T25 flasks in RPMI-1640 culture medium containing 20% fetal calf serum (FCS), 100 mg/mL epidermal growth factor, 2 mmol/l, L-glutamine, 20 mg/mL heparin, 40 mU/mL insulin, 100 U/mL penicillin, and 100 mg/mL streptomycin at 37°C with 5% CO_2_ and 90-95% relative humidity.

###  Peptide simulation

####  Structure preparation and molecular docking

 The determination of a protein’s 3D structure is essential in understanding biological function.^40,41^ Protein Data Bank (PDB) is a well-known database that contains archives of protein structures. Using the PDB database, the initial structure of LRP was obtained (PDB ID: 3V6O). Molecular docking can predict the binding orientation and affinity of target ligand molecules with cognate receptors. To this end, the physical interaction between the LRP and two leptin peptides, Lep 30 and LDP 14, was carried out using the HDock server. This system is a web-based platform for monitoring protein-ligand docking with an optimized algorithm to predict the most favorable binding state. In the next step, the interactions between the candidate peptides and LRP were assessed using the PDBsum server to study the molecular mechanisms underlying the binding process.^42^

####  Molecular dynamics (MD)

 MD simulation is a computational technique used to study the behavior of molecules over time.^43^ In MD simulation, Newton’s equations of motion are considered numerically to determine the movement of atoms and molecules in a target system.^44^ GROMACS (Ver. 2022) is a widely used software package for MD simulations that supports tools for system setup, energy minimization, equilibration, and production runs.^45^ GROMACS employs force fields to describe the interactions between atoms and molecules and calculate the energy of the system at each step of the simulation. MD simulation with GROMACS can provide valuable insights into the structural and dynamical properties of complex biomolecular systems, such as proteins and nucleic acids, and their interactions with ligands or other biomolecules.^46^

####  Peptides labeling

 To track the internalization of LDP 14 and Lep 30, circular SRL (SRLC), Linear SRL (LSRL), and TGN, all the peptides were labeled with fluorescein dye in methanol at pH = 8, for 24 hours. To develop LDP 14 and Lep 30 in NP forms, the PAMAM was conjugated with fluorescein dye at a molar ratio of 1:25 (dendrimer: dye) and dissolved in a 4 mL methanol solution. The exposure of the mixture to alkaline conditions leads to exposure of the NH_2_ terminal groups and reaction with fluorescein dye.^47^ In the next step, peptides were attached to the surface of labeled dendrimers in two ratios (8 and 20) using bifunctional PEG. The mixtures were stirred overnight at room temperature in a dark place. After evaporation of the solvent, the remained products were dissolved in water and dialyzed for 24 hours in a dark place to remove unreacted fluorescent dye.^48^

###  Quantitative cellular uptake of peptides

 To this end, U87, C6, and hBCEC-D3 cells were plated (2 × 10^5^ cells/well) in 6-well culture plates. At 70%-80% confluence, LDP 14 and Lep 30 peptides were added to the culture medium at different concentrations (5, 10, 20, 40, 80, and 100 ppm) and cells were incubated for the next 4 hours. After that, cells were carefully washed with pre-warmed PBS and detached using Trypsin-EDTA solution for flow cytometry analysis using the FACSCalibur system. In this study, a negative control group using 100 nM superhuman leptin antagonist (SHLA) was also included. To assess the temperature-mediated transcytosis of target peptides, the incubation process was done in a culture medium containing 80 ppm LDP 14 and Lep 30 at 4 °C. To evaluate the dual targeting ability of LDP 14, the entry rate of fluorescein-tagged LDP 14 was compared with other peptides such as TGN, Lept30, SRLC, and SRLL in U87 cells using flow cytometry analysis. These analyses were done in triplicate.

###  Synthesis of LDP 14-modified G4 PAMAM dendrimers 

 To investigate the possible negative effect of peptide immobilization on the surface of NPs and targeting capability, the internalization rate of modified LDP 14 and Lep 30 peptides was studied after being attached to the surface of nanocarrier G4 PAMAM dendrimers. In this study, G4 PAMAM dendrimer NPs were synthesized using the Michael addition method.^49^ In short, PAMAM derivatives were fabricated using previous published protocols.^48,50^ PAMAMs with molar ratios of 1:8 and 1:20 were dissolved in PBS (pH 8.0) and reacted to NHS-PEG2000-maleimide (MAL) for 24 hours at room temperature. It is suggested that PAMAM dendrimer amine groups can interact with NHS groups of bifunctional PEG.^48^ To eliminate unreacted PEGs and purify PEG-PAMAM conjugates, the mixture was poured into the dialysis bag (cut of 12-14 kDa). The procedure was continued by dissolving PAMAM-PEG conjugates in PBS (pH 7.0). The conjugates were reacted with LDP 14 at the same molar ratio of PEG at room temperature for 24 hours. Herein, the MAL groups of the attached PEG molecules can react with the SH group of the terminal cysteine. To fabricate fluorescent PAMAM-PEG-LDP 14 NPs, fluorescein-labeled PAMAM–PEG–LDP 14 and fluorescein-labeled PAMAM were prepared by reacting fluorescein in 100 mM NaHCO3 for 12 hours kept under light protection. Purification of labeled NPs was done by bag dialysis.

###  Characterization of LDP 14-modified dendrimers and their derivatives

 The chemical bond formation synthesized NPs (PAMAM, PAMAM-PEG, and PAMAM-PEG-LDP 14 were analyzed by FTIR and NMR. The hydrodynamic diameter and zeta potential values were determined using dynamic light scattering (DLS). Transmission electron microscopy (TEM) was used to analyze the morphology and real size of NPs (PAMAM-PEG-LDP 14). To monitor the changes in molecular weight, SDS-PAGE electrophoresis was used after the fabrication of NPs. According to our previous experience, 18% acrylamide gel at 120 V for 120 minutes can efficiently separate dendrimer derivatives. A suitable N/P ratio was checked by the agarose gel retardation assay to monitor polyplexes formation and then characterized by DLS.

###  Cellular uptake of PAMAM derivatives

 To see whether the targeting efficacy of ligands is changed after attaching to the surface of NPs, the internalization of LDP 14 was examined following immobilizing onto the dendrimers. In short, 2 × 10^5^ U87 cells were seeded in 6-well culture plates. After 48 hours, cells were incubated with 2.5, 5, 10, and 20 ppm of fluorescein-labeled PAMAM-PEG_(20)_, PAMAM-PEG-LDP 14_(20)_, and PAMAM-PEG-LDP 14_(8)_ for 4 hours. After that, cells were rinsed with cold PBS three times to remove the free NPs in the supernatant. The percent of fluorescent-positive cells was quantitatively checked using flow cytometry. In this study, 2% FBS was used to prevent possible unwanted effects of PC-modified peptide-NP conjugates.^51^ Three sets of experiments were performed.

###  Gene-carrying capability of LDP 14-PAMAM conjugate

 To monitor the gene-carrying capability of developed NPs, ethidium bromide (EBr)-labeled pEGFP-N1 plasmid was used as a reporter. To this end, we incubated 20 µg of the plasmid with 100 mM EBr for 24 hours in a dark place. The best ratio of the abovementioned NPs (PAMAM–PEG–LDP 14) to plasmid (N/P) was determined using 5:1, 8:1, 10:1, 12:1, and 20:1 ratios (w/w) with a focus on polyplexes formation and stability in agarose retardation assay.

###  Internalization of PAMAM-PEG-LDP 14/pEGFP-N1 complexes 

 U87 cells were cultured in 6-well plates and incubated with polyplexes in a 10:1 N/P ratio containing 2 and 4 µg plasmid for 4 hours. To indicate the leptin-mediate entry of polyplexes, U87 cells were incubated with EtBr-labeled PAMAM–PEG–LDP 14/pEGFP-N1 NPs in the presence of 100 nM SHLA. After the completion of incubation time, cells were washed with cold PBS (pH 7.4) and observed using a fluorescent microscope (Olympus, Japan). The cellular uptake was analyzed using flow cytometry as above-mentioned. This assay was performed in triplicate.

###  The effect of PC formation on NP internalization

####  Blood collection and PC formation assay

 A total number of 6 blood samples were collected from healthy male volunteers (aged between 25 to 30 years old), using an FL medical (Italy) blood collection system containing K_2_EDTA and a protease inhibitor. Plasma samples were separated by centrifugation at 11 000 rpm for 20 minutes. To exclude the person-to-person variation, all isolated plasma samples were pooled and used for PC analysis. Using centrifugation at 2400 rpm, at 4 °C for 4 minutes, remnant cell debris was also eliminated. Then, G4 PAMAM dendrimers, LDP 14-dendrimer, and Lep 30-dendrimer conjugates were incubated with 55% human plasma at 37 °C for 1 hour.^51^ The loosely attached or unattached proteins on the periphery of synthesized NPs were eliminated with three-time PBS washes and centrifugation at 10 000 rpm for 30 minutes. Finally, the PC-coated NPs were dissolved in 500 μL PBS and subjected to different analyses. The composition of the formed PC was qualitatively investigated using the SDS-PAGE electrophoresis.

 To address the role of PC on the targeting ability of the dendrimer derivatives, hBCEC-D3 cells were plated (2 × 10^5^ cells/well) in 6-well plates. After reaching 70%−80% confluency, the culture medium was replaced with a fresh culture medium containing 2% FBS, 150 μg/mL fluorescein-labeled G4 PAMAM dendrimers, LDP 14-dendrimer, and Lep 30-dendrimer conjugates in PC-free and -coated forms. After 4 hours, cells were washed with cold PBS three times, stained with Hoechst 33342, and visualized using a fluorescence microscope (Olympus, Japan). The fluorescence intensity and cellular uptake of NPs were quantitatively analyzed using a BD FACSCalibur flow cytometer and FlowJo software (Ver. 10). In this study, the live cells were gated for fluorescence intensity analyses.

####  Separation of retrieved PC proteins on NP surface using SDS-PAGE 

 Plasma-treated NPs were boiled in the loading buffer at 100 °C for 5 minutes. 10 μL of each sample was electrophoresed using 10% SDS-PAGE at 120 V for 100 minutes. Then, gels were rinsed in double-distilled water (ddH2O) and fixed over 24 hours in a de-staining solution (MeOH 50% in ddH2O) with gentle agitation at room temperature. Image acquisition was done using the Gel doc equipment (Vilber Lourmat, France). The presence or absence of protein bands and their intensities (area under the curve, AUC)) were calculated using ImageJ software.

###  In vivo imaging analysis

 Around 4–5-week-old male Balb/c mice (n = 6), weighing 20–25 g, were purchased from Razi Vaccine and Serum Research Institute (Karaj, Iran). The animal was kept in standard cages with unrestricted access to food and water under in standard 12-hour light/dark cycles. Animals were treated according to Guide for the Care and Use of Laboratory Animals (NIH, 1986), and all phases of this study were approved by the Local Ethics Committee of Tabriz University of Medical Sciences. To assess the BBB transfer of CS-PEG-CDX/pEGF into the brain, polyplexes containing pEGFP (10:1, CS-PEG-CDX to DNA, w/w) with a dose of 50 µg DNA/mice were injected intraperitoneally once a day for 5 consecutive days. After this period, the mice were imaged using Tehran University of Medical Sciences Preclinical Core Facility CRi Maestro (Fluorescence) Imaging (Kodak Fx Pro, New York, USA).^18^

###  Statistical analysis

 Data were analyzed using one-way ANOVA with Tukey post hoc. *P* values below 0.05 were considered statistically significant. Three sets of experiments were performed otherwise mentioned.

## Results

###  Molecular docking and dynamics

**Figure 1:**
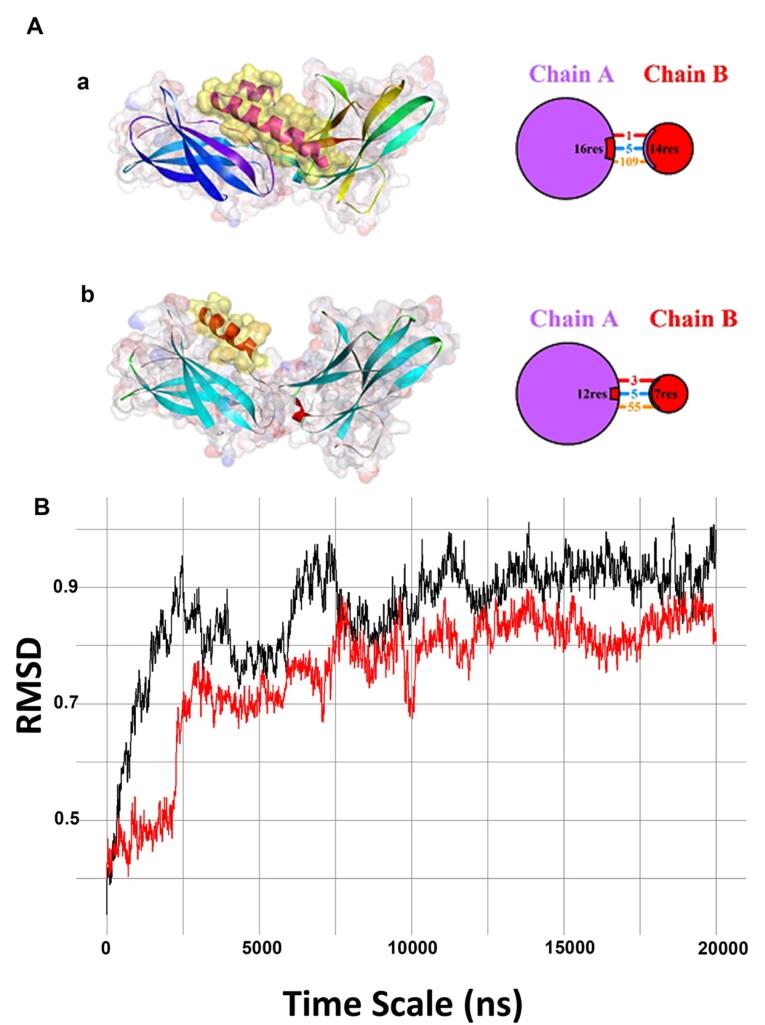
A) a. The interaction of Lep 30 peptide with ObR displayed 5 hydrogen bonds (blue -) and 1 salt bridge (red -). b. The interaction of the LDP 14 peptide with ObR is done via 5 hydrogen bonds (blue -) and 3 salt bridges (red -). B) In MD simulations, RMSD is often used as a stability index of a biomolecule or ligand-protein complex. The RMSD value quantifies the deviation of atomic positions of a biomolecule or complex throughout simulation time. In this context, the comparison of RMSD values at the 20 (ns) 1(Å) mark indicates that the LDP 14 peptide exhibits a higher degree of stability compared to other peptides or biomolecules that were simulated

 Molecular docking analysis revealed a higher docking score in the interaction of Lep 30 peptide with ObR, making it a promising candidate for drug delivery purposes (Table 1). It was suggested that the number of hydrogen and salt bridge bonds formed by the peptides is important in reciprocal physical interactions.^52,53^ Interestingly, despite the lower docking score, the LDP 14 peptide formed a staggering five hydrogen bonds and three salt bridge bonds with ObR, making it superior to Lep 30 in terms of peptide-ObR affinity (Figure 1Aa-b). These features indicate that interaction between peptides and receptors is not solely limited to the docking scores but also complex molecular mechanisms. It is thought that these data can open up new avenues of research in the field of pharmaceutical research and the development of novel treatments for various pathological conditions. MD simulations indicated significant insights into the stability and potential superiority of the LDP 14 and Lep 30 peptides. More importantly, the root means square deviation (RMSD) analysis showed that the LDP 14 has significantly better stability compared to the Lep 30 (Figure 1B). Based on these findings, the use of multi-dimensional approaches, including stability and molecular docking, is mandatory in peptide analysis. Future research in this field holds great promise for unlocking new advancements in drug development.

**Table 1:** Comparing docking scores and ObR interactivity for Lep 30 and LDP 14 peptides.

**Rank (Lep 30)**	**1**	**2**	**3**	**4**	**5**
Docking score	-206.96	-204.39	-203.26	-200.95	-196.4
Confidence score	0.7575	0.748	0.7437	0.7348	0.7167
**Rank (LDP 14)**	**1**	**2**	**3**	**4**	**5**
Docking score	-177.75	-168.65	-167.93	-166.8	-160.51
Confidence score	0.6353	0.5922	0.5887	0.5832	0.5524

###  Cellular uptake of LDP 14

**Figure 2:**
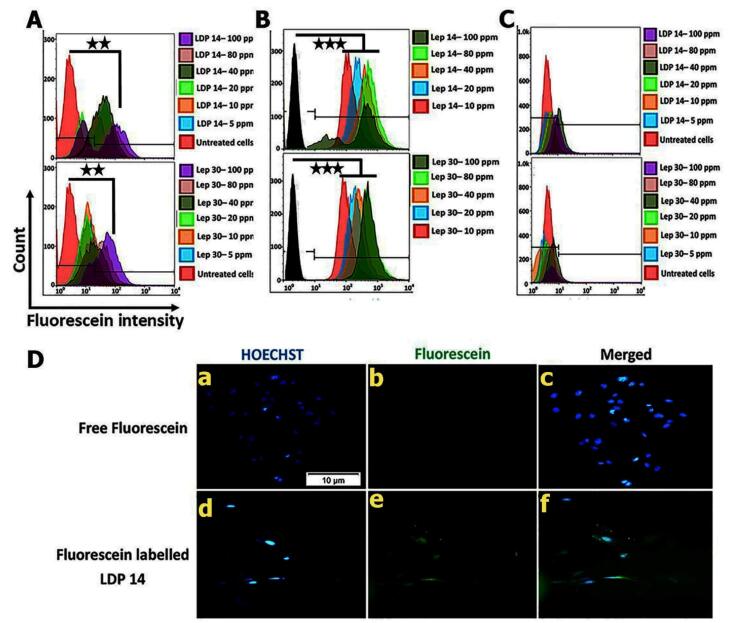
Quantitative analysis of cellular uptake of LDP 14 and Lep 30 in A) hBCEC-D3, B) human U87 glioma, and C) rat C6 glioma cell lines using flow cytometry analysis after 4 hours incubation time. E) fluorescence images of LDP 14 internalization by rat C6 glioma cells at a concentration of 100 ppm (d-f) against free fluorescein dye (a-c). Scale bar: 10 µm, Blue: Hoechst, Green: Fluorescein, (n = 3) **P < 0.01; ***P < 0.001; ****P < 0.0001

 The validity of *in silico* data was re-checked by monitoring the targeting ability of the modified LDP 14 and Lep 30 peptides on ObR. To this end, three ObRs expressing cell lines were used in *in vitro* conditions. Lep 30 was used as the best-known leptin-derived peptide which can identify the ObRs on the hBCEC/D3 for passing the therapeutics into the brain parenchyma. Flow cytometry results indicated that the percentage of the fluorescent cells was higher in LDP 14 groups at all treated doses except 5 ppm compared to Lep 30-treated cells. As shown in Figure 2A, the mean fluorescent intensity (MFI) of the hBCECs incubated with LDP 14 is higher than Lep 30 (*P* ≤ 0.01). Data confirmed that by increasing the concentrations of peptides to 40 ppm, the percent of fluorescein-labeled cells was increased in hBCEC-D3 and U87 glioma cells, and these values were more at a dose of 100 ppm. Based on the data, 40 ppm labeled LDP 14 can enter the hBCEC-D3 and U87 glioma cells more than the labeled Lep 30 at the same concentration (Figure 2A and B) (*P* ≤ 0.001). Noteworthy, the MFI of LDP 14 treated hBCEC-D3 cells (Figure 2A) was more than Lep 30-treated cells in a concentration-dependent manner, especially from 40 ppm (*P* ≤ 0.05). In contrast, the incubation of rat C6 glioma cells with different doses of LDP 14 and Lep 30 did not yield a fluorescein-labeled cell population compared to the non-treated cells. These data indicate that LDP 14 exhibits more on-target delivery properties compared to Lep 30 in ObRs expressing cells in a species-dependent manner (human-specific activity).

 In this study, we aimed to apply a sophisticated delivery platform targeting the glioma cells inside the brain tissue. Qualitative and quantitative fluorescence analysis indicated labeled-Lep 30 and –LDP 14 were internalized by human U87 glioma cells and nearly 100% of treated cells became fluorescent after incubation time.^54^ In the flow cytometry panel, we found that the MFI values were higher in U87 glioma cells as compared to hBCECs (*P* ≤ 0.001) (Figure 2A and B). The higher internalization of LDP 14 in human U87 cells can be associated with the higher expression of short isoform leptin receptors in U87 cells, compared to h-BCEC-D3 cells.^55-58^

**Figure 3:**
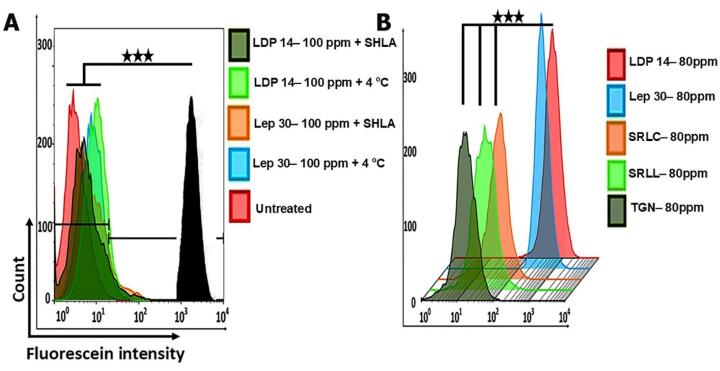
Quantitative internalization rate of LDP 14 and Lep 30 by hBCEC-D3 cells at 4°C and, in the presence of SHLA (A), quantitative comparison between LDP 14 and Lep 30, SRLC, SRLL, and TGN internalization by U87 cell line (B). (n = 3) **P < 0.01; ***P < 0.001; ****P < 0.0001

 Surprisingly, a different uptake pattern was indicated in rat C6 glioma cells in which LDP 14 even at concentrations below 100 ppm were negligibly internalized compared to Lep 30 which was not significant (Figure 2C). Numerous studies have shown the overexpression of ObRs in rat C6 glioma cells.^59-61^ Consistent with data from our *in silico* analysis demonstrated that the LBD of ObRs differs between humans/mouse and rats. In support of this notion, different recombinant ObR inhibitors like SHLA and SMLA are used in humans and mice. Interestingly our data showed even a high dose (100 ppm) of labeled LDP 14 could not be internalized by rat C6 glioma cells (Figure 2D). As above-mentioned, these data indicate the species specificity of the designed peptide. In this study, human U87 glioma cells were selected for subsequent analyses due to higher cellular uptake of LDP 14. To assess whether the developed peptide can enter the hBCEC-D3 cells by a selective interaction with ObRs, we studied ObR-mediated uptake of LDP 14 in the presence of ObRs inhibitor namely SHLA using flow cytometry analysis. Also, to investigate the ATP-dependent transcytosis internalization of LDP 14, cells were treated with 100 ppm, fluorescein-LDP 14 at 4 °C for 4 hours. As expected, data showed the involvement of ObR in the LDP 14 internalization into the hBCEC-D3 cells in an ATP-dependent manner (*P* ≤ 0.001) (Figure 3A). Lep 30, a well-known leptin-derived peptide, was used as a control in this experiment. Similarly, the co-treatment of human U87 cells with SHLA and LDP 14 exhibited similar results (Figure 3A).

 In addition to the targeting effect of LDP 14 on brain endothelial cells, the interaction of this peptide with human U87 glioma cells was also assessed in comparison with four well-known BBB targeting peptides including SRLL, SRLC, TGN, and Lep 30. Interestingly, data proved the higher dual targeting property of LDP 14 in the human U87 glioma more than SRLL, SRLC, TGN, and even Lep 30 (*P* ≤ 0.001) (Figure 3B).

###  NPs Characterization

####  FTIR

**Figure 4:**
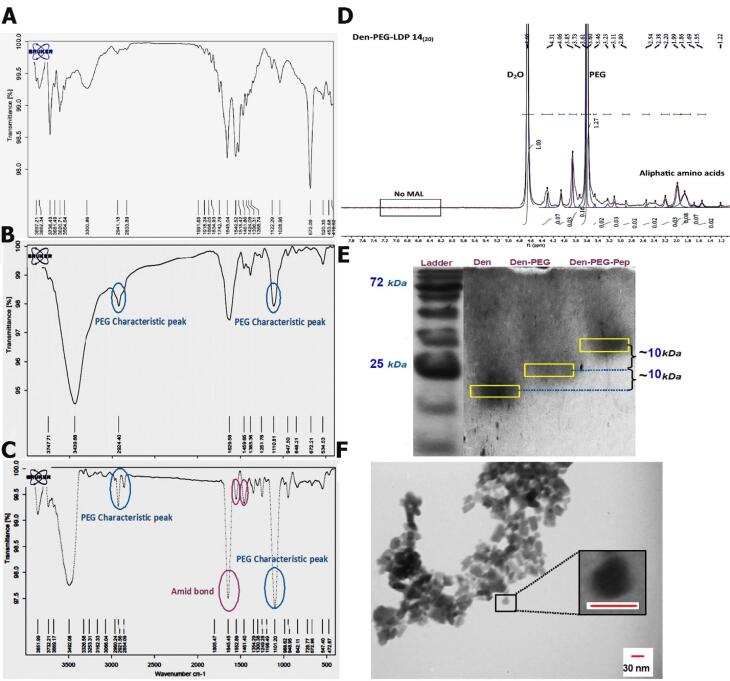
FTIR spectra of A) G4 PAMAM dendrimers, B) Den-PEG, C) Den-PEG-LDP 14. D) Final NMR spectrum of Den-PEG-LDP 14. E) SDS-PAGE of Den, Den-PEG, and Den-PEG-LDP 14. F) TEM image of Den-PEG-LDP 14 NPs. Den: dendrimers; Scale bar: 30 nm

 The FT-IR spectra of G4 PAMAM dendrimers, dendrimer-PEG, and dendrimer-PEG-LDP 14 conjugates are indicated in Figure 4A-C. The tensile vibration of the N–H bond is related to the G4 PAMAM was observed at 3441 cm^-1^. Characteristic peaks of PEG molecules appear at 2924 and 1110.8 cm^-1^ after PEG attachment. The increase in the intensity of C–H tensile vibration peaks at 1459 and 947 cm^-1^ are related to the repeated units of (CH and CH2) in the PEG structure. Following the reaction of the LDP 14 peptide with den-PEG polymers, the peak at 1629 cm^-1^ was replaced with peaks at 1645, 1552, and 1461 cm^-1^ associated with amide bonds I to III, respectively.

####  NMR

 In the NMR we showed the spectrum of finalized NPs (Figure 4D), the solvent peak D2O was observed at 4.78 ppm. The methylene protons of PAMAM branch units between 2.0-3.2 ppm were relatively diminished after the addition of peptide to the dendrimer solution.^62^ Data illustrated that the MAL group of the PEG molecules was eliminated in 6.1 ppm following the attachment of the LDP 14 peptide to the dendrimer-PEG polymers. Also, the peak at 3.7 ppm is related to the repetitive units of PEG, indicating bifunctional PEG linkers in the synthesized polymer structures.

####  SDS-PAGE

 SDS-PAGE technique is a fast and repeatable low-cost method to study the molecular weights of proteins and aminated chemical compounds.^63,64^ Here, we used the SDS-PAGE technique for analyzing the Den-PEG and Den-PEG-peptide formation. Due to the different MW of the dendrimer, Den-PEG, and Den-PEG-Peptide complexes, it is suggested that almost the near half of PEG and peptides were attached to the terminal amines of PAMAM dendrimers (Figure 4E). Due to the relatively similar MW of bifunctional PEG and LDP 14 peptide, results showed that almost half of the nominal numbers of PEG and peptides (8 and 20) were attached to the dendrimers. To the best of our knowledge, this is the first time that SDS-PAGE was used for MW of modified dendrimers.

####  DLS

 DLS was performed to measure the hydrodynamic diameter and zeta potential of the different synthesized NPs. Table 2 shows the alterations in the size and zeta potential of naked G4 dendrimers, Den-PEG, Den-PEG-LDP 14, and Den-PEG-LDP 14/pEGFP-N1 conjugates, respectively. As shown in Table 2, the hydrodynamic diameter of the G4 dendrimers increases with attaching bifunctional linker, peptides, and plasmid, while the zeta potential values are changed based on the cargo size and ratio of attached agents to nanoparticles.

**Table 2:** Physicochemical properties of the G4 PAMAM dendrimer derivatives

**Formulation**	**Process Yield**	**Particle size (nm)**	**Zeta potential (mV)**
		**DLS TEM**	
Dendrimer	80.63	4.8 ± 0.5 NA	+ 24.3 ± 5.45
Dendrimer-PEG	50	21.06 ± 3.2 NA	-2.17 ± 3.45
Dendrimer-PEG-LDP 14	100	65.14 ± 17.87 30	-8.65 ± 4.15
Dendrimer-PEG-LDP 14/pEGFP-N1; N/P = 10:1	100	195 ± 68.1 NA	-7.13 ± 4.44

NA: not assessed.

###  Cellular uptake of NPs by U87 cells 

**Figure 5:**
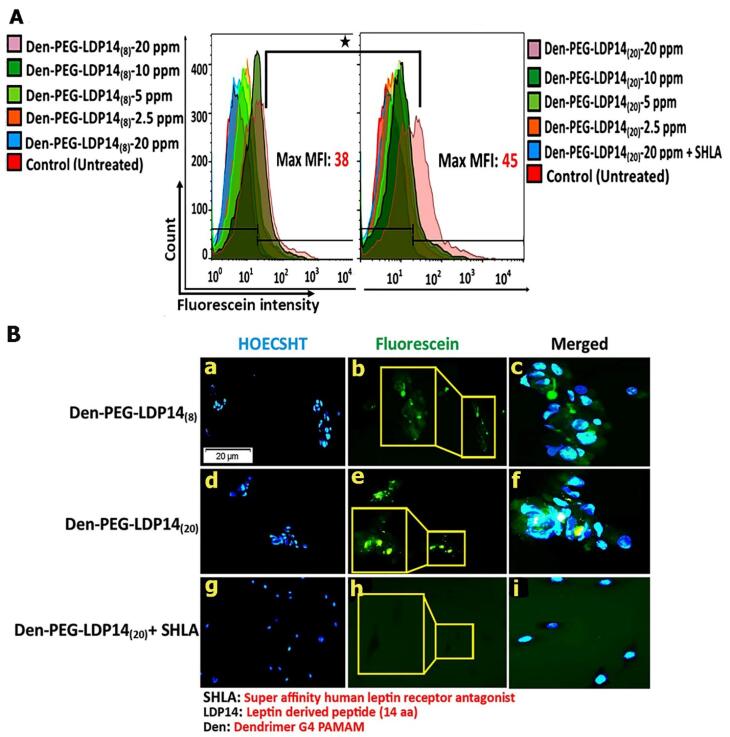
Cellular internalization of A) fluorescein-labeled Den-PEG_(8)_-LDP 14, Den-PEG_(20)_-LDP 14 by U87 glioma cells in different concentrations in the presence and absence of SHLA. B) Qualitative cellular uptake of fluorescein-labeled Den-PEG_(8)_-LDP 14 (a-c) and fluorescein-labeled Den-PEG_(20)_-LDP 14 by U87 cells in the presence (g-i) and absence of SHLA (d-f). Scale bar: 20 µm, Blue: Hoechst, Green: Fluoresacein, (n = 3) **P < 0.01; ***P < 0.001; ****P < 0.0001

 Following the dual targeting ability of LDP 14 in targeting h-BCEC-D3 and U87 cell lines more efficiently than Lep 30, we decided to investigate the targeting of LDP 14 after attachment onto the surface of dendrimers for future *in vivo* studies. To this end, U87 cells were incubated with low concentrations of labeled fluorescein-labeled PAMAM-PEG-LDP 14 NPs to investigate cellular uptake using flow cytometry and a fluorescent microscope quantitatively and qualitatively, respectively. Again, SHLA was used to inhibit the activity of ObRs. For quantitative analysis, cells were incubated with 2.5, 5, 10, and 20 ppm PAMAM-PEG-LDP 14 NPs and analyzed using flow cytometry (Figure 5A). Flow cytometry analysis and fluorescent microscopy revealed dose-dependent uptake of synthesized NPs (Figure 5A). To be specific, by increasing the concentration of LDP 14 un the surface of NPs, the MFI (38 for LDP 14_(8)_ VS 45 for LDP 14_(20)_) and percent of labeled cells were increased, indicating a close relationship between the peptide density and internalization rate (*P* ≤ 0.05) (Figure 5A). More importantly, qualitative and quantitative analysis of LDP 14_(20)_-modified NPs uptake proved that SHLA remarkably reduced the uptake of NPs closed near-to-control levels (*P* ≤ 0.05) (Figure 5A and B). As can be seen in Figure 5B(g-i), there is no fluorescent signal inside the cells even by increasing the UV exposure time in the fluorescent microscope, and only the ghost-like shape of cells appeared.

###  Characterization and cellular uptake of LDP 14-PEG-PAMAM/PEGFP-N1 polyplexes

 According to the studies, it has been determined that the final size of the nanoparticle/cargo has an inevitable role in the delivery of the therapeutics to the diseased site. To evaluate the effect of the nature of therapeutic cargo on the targeting capability of LDP 14-modified dendrimers, LDP 14-modified dendrimers/pEGFP-N1 polyplexes were synthesized and characterized using different techniques. It should be considered that the zeta potential of synthesized nanoparticles rises by increasing the ratio of N/P which results in a positive charge of polyplexes. Results of 0.7% agarose gel demonstrate proper stability of polyplexes in N/P = 10 (Supplementary file 1, Figure S1). The size and zeta potential of LDP 14-PEG-PAMAM /PEGFP-N1 complexes are 196 nm and -7 mV respectively (Supplementary file 1, Figure S1). It is worth mentioning that the positive charge of nanoparticles improves the interaction of nanoparticles with cellular membranes which leads to higher recognition of ligands by expressed receptors.

**Figure 6:**
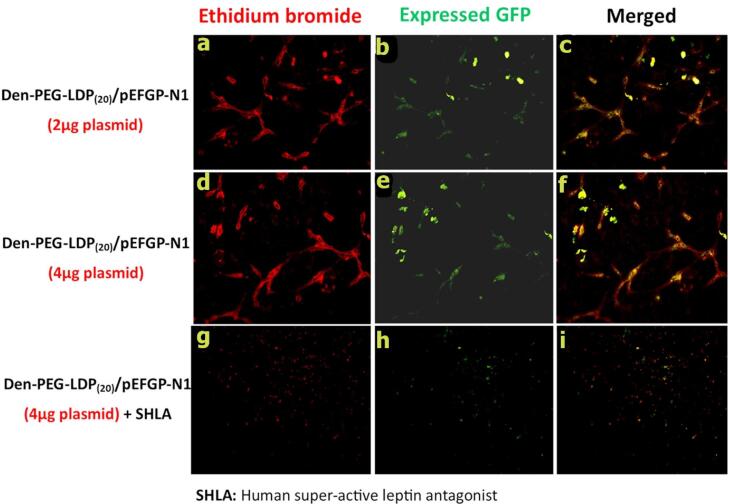
Qualitative uptake of Den-PEG_(20)_-LDP 14/pEGFP-N1 polyplexes in concentration (a-c) (d-f) and ObR-dependent manner (g-i). Scale bar: 20 µm, Red: Ethidium bromide, Green: Green fluorescent protein

 Cellular uptake of LDP14-PEG-PAMAM/pEGFP-N1 nanocomplexes was qualitatively and quantitatively studied in the U87 glioma cell line using a fluorescence microscope and flow cytometry, respectively. As shown in Figure 6, cellular uptake and plasmid concentration are directly related to each other. After 72 hours of incubation, GFP (green fluorescent protein) expression is traceable by fluorescent microscopy. Quantitative analysis by flow cytometry technique demonstrates higher cellular uptake of 40 ppm dendrimer (MFI: 55 for 20 ppm VS 62 for 40 ppm) containing polyplexes compared with 20 ppm (Figure 6, n = 3, *P* value ≤ 0.05,Supplementary file 1, Figure S2**)**. Interestingly, the presence of SHLA as ObR inhibitors suppressed the uptake of polyplexes (Figure 6, n = 3, *P* value ≤ 0.001,Supplementary file 1, Figure S2).

**Figure 7:**
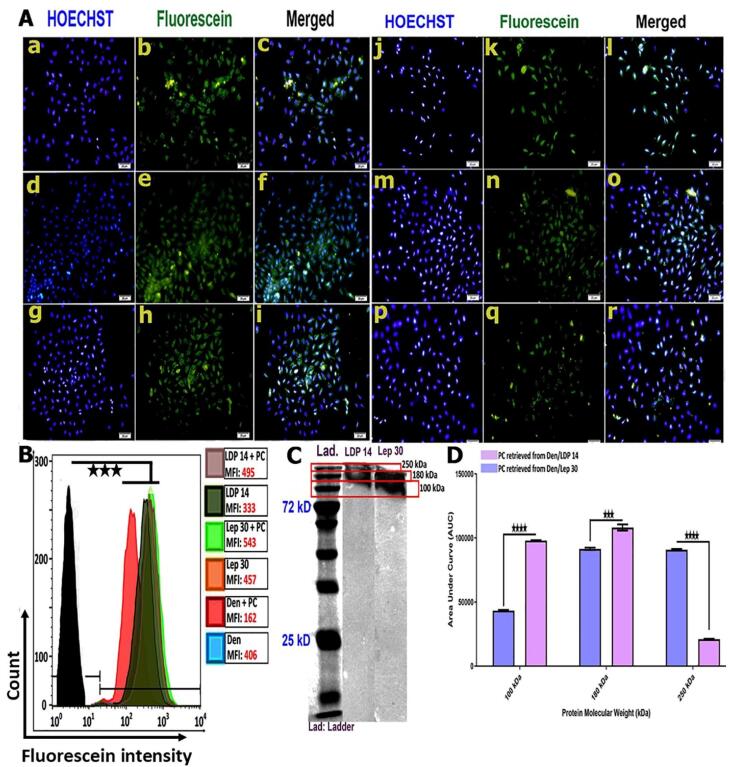
A) Qualitative uptake of Den, Den-PEG-LDP 14, and Den-PEG-Lep 30 in the presence and absence of PC formation after incubation with healthy human plasma. Den (a-c), Den + PC (d-f), Lep 30 + PC (g-i), Lep 30 (j-l), LDP 14 + PC (m-o) and LDP 14 (p-r). B) Quantitative uptake of Den, Den-PEG-LDP 14, and Den-PEG-Lep 30 in the presence and absence of PC. Scale bar: 20 µm, Blue: Hoechst, Green: Fluoresacein, (n = 3) **P< 0.01; ***P < 0.001; ****P < 0.0001. C) SDS-PAGE of PC retrieved from LDP 14 and Lep 30 modified dendrimers. D) Quantitative analysis of retrieved proteins from Den-LDP 14 and Den-Lep 30. (n = 6), **P < 0.01; ***P < 0.001; ****P < 0.0001

 According to results, LDP 14 with 16 amino acids less than Lep 30 has been shown to better function as a dual targeting ligand, up to here. But, the final property of a ligand which in recent years turned into an important matter in targeted drug delivery^65,66^ is still unanswered, which is the effect of PC on the targeting efficiency of LDP 14-modified NPs. Surprisingly, the results of SDS-PAGE illustrated that Lep 30 in this experiment works better than LDP 14, because not only more PC formation from healthy volunteers had no negative effect on their targeting efficiency but enhanced it significantly Figure 7Aa-f and B. Flow cytometry results showed that PC formation remarkably reduces the uptake of dendrimers (*P* ≤ 0.05) (Figure 7Aa-f and B). Also, Lep 30-modified dendrimers after PC formation show better uptake than LDP 14-modified dendrimers (*P* ≤ 0.05) (Figure 7Aj-l and B). Interestingly, the difference between the internalization efficacy of LDP 14-modified dendrimers + PC with LDP 14-modified dendrimers - PC was higher than their Lep 30 counterpart (*P*≤ 0.01) (Figure 7Am-r and B). This finding would be due to different proteins that LDP 14 absorbed compared to Lep 30, which enhance their internalization efficacy. In this regard, retrieved proteins from LDP 14 and Lep 30-modified dendrimers proved this claim (*P* ≤ 0.001) (Figure 7C and D). As shown in Figure 7 panels C and D, MW of the proteins absorbed onto the surface of LDP 14-modified dendrimers are more than 180 kDa, while in the case of Lep 30-modified dendrimers are 100-180 kDa. Based on these findings, strongly suggested that the exact types of absorbed proteins Nano-LC mass apparatus would be helpful for future directions.

###  In vivo imaging analysis

**Figure 8:**
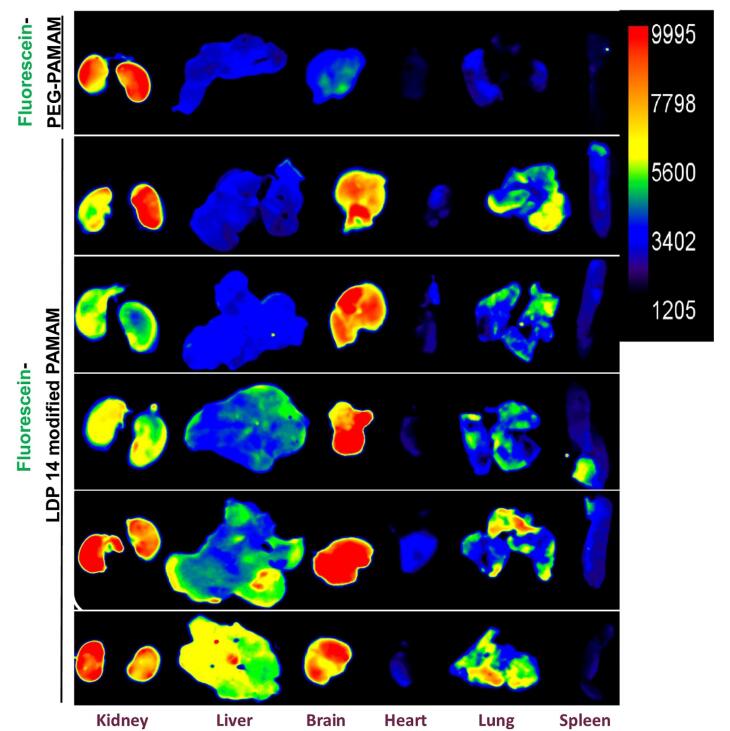
Qualitative ex vivo imaging of major organs of the mice were treated with fluorescein labeled LDP 14-modified G4 PAMAM dendrimer (n = 5) and fluorescein labeled PEG-modified G4 PAMAM dendrimer fluorescein (n = 3) (control). Images were taken 8 h after IP injection of NPs

 For evaluating the efficiency of synthesized nanoparticles to reach the brain, the fluorescein labeled LDP 14-modified G4 PAMAM dendrimers and fluorescein labeled PEG-modified G4 PAMAM dendrimers (as a control) were injected intraperitoneally at a 200 μL total volume injection (80 μM dendrimer) per mouse once a day.^18^ To assess the biodistribution of NPs, different organs including the heart, spleen, kidneys, and liver were sampled along with the brain. As shown in Figure 8, a strong signal can be seen in the brain of the animal that received labeled LDP 14-modified G4 PAMAM dendrimers compared to the control group. The biodistribution of nanoparticles in vital organs like the liver, spleen, heart, and kidneys was also evident in which the kidney was the main target for the mentioned NPs (Figure 8).

## Discussion

 In recent years, some peptides like Angiopep-2 have been used as targeting ligands for drug delivery into the human brain in different clinical trials.^67,68^ Among different kinds of ligands, only limited numbers exhibited dual targeting moieties.^69^ It was suggested that dual targeting behavior completely depends on the expression level of specific receptors on the BBB cells and pathological sites. For example, low-density lipoprotein receptors are expressed on the BBB interface and glioma cells.^70^ Peptide ligands derived from natural sources are at the center because of their appropriate biological properties.^71^ In this regard, leptin-derived peptides such as Lep 30 and cognate ObRs are commonly used as shuttle machinery for crossing therapeutics through the BBB interface.^72^ Upon the attachment of ligands with different kinetics to ObRs, the transcytosis phenomenon is activated in BBB cells.^73,74^ Commensurate with these descriptions, finding the highly specific ligands with maximum internalization capacity is at the center of attention in brain pathologies. Here, a novel leptin hormone peptide was identified based on bioinformatic prediction and MD simulation, and cellular uptake was examined in vitro on human endothelial and glioma cells.

 Bioinformatic analysis revealed that the identified peptide LDP 14 can recognize the short isoform of ObR as compared to Lep 30, the most efficient leptin-derived peptide.^28^ We also noticed that of the total of 30 amino acids that existed in the structure of Lep 30, about 16 amino acids did not have an efficient role in the stability of the peptide-receptor interaction. Preliminary docking data showed a higher affinity of Lep 30 to ObRs compared to LDP 14. Of note, consistent with MD simulation and *in vitro* data, LDP 14 internalized to the hBCEC-D3 cells and human U87 glioma cells more effectively than Lep 30. It seems that these effects are associated with the alteration in the affinity of LDP 14 to the shot isoform receptors resulting in the recycling and relaxation time of the receptor after attaching to the peptide.^75,76^ The high rate of Lep 30 and LDP 14 internalization in human U87 glioma cells indicates the overexpression of ObRs in these cells.^54^ To validate the specificity of the designed peptide in human cells, we also incubated the rat C6 glioma cells labeled LDP 14. Both *in silico* data and *in vitro* analyses confirmed the lack of LDP 14 internalization in rat glioma cells, indicating species specificity of the used peptide. The difference in internalization rate would be related to ObR expression rate, and varied protein structure of ObR in humans compared to rat counterparts, limiting the physical interaction.

 In terms of NP structure, the attachment of LDP 14 on the surface of G4 PAMAM NPs did not alter peptide targeting efficacy. To be specific, immobilization of LDP 14 on PAMAM NPs did not alter the conformational structure of LDP 14 in a way to its reduced interaction with ObR. Because the size and physicochemical properties of particles can affect the delivery efficiency, the gene-carrying capability of LDP 14-PAMAM conjugate was examined. In this regard, the LDP 14-PAMAM conjugate with GFP-expressing plasmids was added to the culture medium. Previous data have revealed that gene delivery approaches should be able to circumvent the lysosomal degradation system and preserve the genetic cargo.^77^ In most delivery routes based on endocytosis, the activation of lysosomes in the next steps can neutralize the genetic elements.^78^ Here, certain NP structure was selected for delivery approaches to escape the endosomal system along with ligand (LDP 14) that has receptor (ObR) with prominent expression rate and minimum effects on lysosomal degradation in caveolae independent and/or caveolae-mediated endocytosis.^79^ Data revealed that human U87 glioma cells were efficiently transfected with Den-LDP 14/pEGFP-N1 polyplexes, indicating the suitability of the designed platform for delivery approaches in brain cancers.

 In the next step, we analyzed the interfering role of PC formation on designed NPs. The physicochemical phenomenon in biological fluids is affected by the size and surface signature of NPs.^80^ In our previously published article, we indicated that folic acid-modified chitosan NPs decorated with opsonin proteins (e.g. IgM) can accelerate NP elimination by the activation of reticuloendothelial phagocytes (splenic and hepatic macrophages).^51^ We believe that this experiment is a pioneer study in addressing the possible interfering role of PC in the internalization of LDP 14-dendrimer and Lep 30-dendrimer conjugates by hBCEC-D3 cells. SDS-PAGE data confirmed that the molecular weights of conjugates were increased after incubation with human plasma samples. However, the decoration of designed NP structures with PC did not alter the internalization properties of the target cells. As finding the *in vivo* biodistribution is the essential step in characterization of functionalized NPs, we evaluated the biodistributation of fluorescein labeled LDP 14-modified G4 PAMAM dendrimers and fluorescein labeled PEG-modified G4 PAMAM dendrimers after injection to the mice. In contrast to findings that naked PAMAM dendrimers accumulated in the spleen of the mice due to adsorption of IgM unto their surface,^81,82^ 8 hours after injection our PEGylated PAMAM dendrimers have been located at kidneys without any significant signal in the spleen and the liver. The most relevant reason behind this result is the lower size of NPs than pores found in the endothelial cells of kidney fenestrated capillaries. More importantly, fluorescein labeled LDP 14-modified G4 PAMAM dendrimers successfully reached the brain of the mice, while except to kidney the other vital organs did not show significant fluorescent signal. According to databases, this happened due to the high homology of ObR between human and mouse compared to rat. Therefore, results of this study showed that LDP 14 can be used as a novel and highly efficient ligand for dug/gene delivery to the brain.

## Conclusion

 Targeting delivery potential and internalization capability of novel leptin hormone-derived peptide, namely LDP 14, was examined on three different cell lines and compared to Lep 30. Our data indicated species specificity of LDP 14 for targeting ObR on the surface of human BBB vascular cells and glioma cells. The selected peptide had no internalization in rat C6 glioma cells. LDP 14-modified G4 PAMAM dendrimers, alone and in complex with pEGFP-N1 plasmid, were successfully applied for efficient delivery to the hBCEC-D3 and human U87 glioma cell lines. In contrast to numerous previously recognized ligands, PC formation not only did not hamper the cellular uptake of LDP 14-modified dendrimers but also this phenomenon increased the internalization rate after being incubated with human plasma samples. In summary, it was noted that the LDP 14 peptide could be considered as a new promising dual targeting peptide for drug/gene delivery to the human brain under pathological conditions such as tumors. Development and application of more sophisticated delivery platforms with higher specificities and affinities to BBB structure, especially the LDP 14-ObR axis, can increase the targeting efficiency into the brain parenchyma during several pathologies.

## Competing Interests

 The authors report no conflicts of interest in this work. The authors declare no competing financial interest.

## Data Availability

 Data will be made available on request.

## Ethical Approval

 All phases of this study were approved by the Local Ethics Committees of the Tabriz University of Medical Sciences (IR.TBZMED.VCR.REC.1400.408).

## Supplementary Files

Supplementary File contains Figure S1 and S2.
