# Supplementary material for: Discovery of a Novel Dual Targeting Peptide for Human Glioma: From In Silico Simulation to Acting as Targeting Ligand
Source: Adv Pharm Bull. 2024 Mar 10;14(2):453–68. doi: 10.34172/apb.2024.033 (PMC11347739; doi:10.34172/apb.2024.033)
Supplement: Supplementary file 1 — Supplementary File contains Figure S1 and S2. [file apb-14-453-s001.pdf]

Adv Pharm Bull, 2024, 14(2), 453-S1
doi: 10.34172/apb.2024.033
https://apb.tbzmed.ac.ir

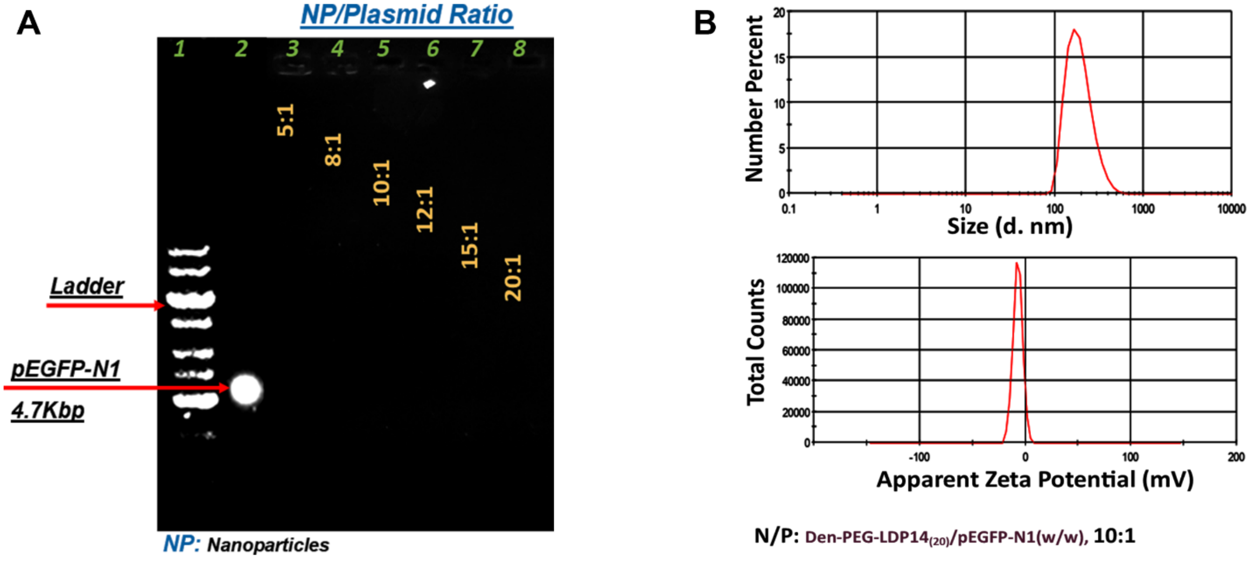

Figure S1. A) Agarose gel electrophoresis of different N/P, B) size and zeta potential of the N/P 10:1.

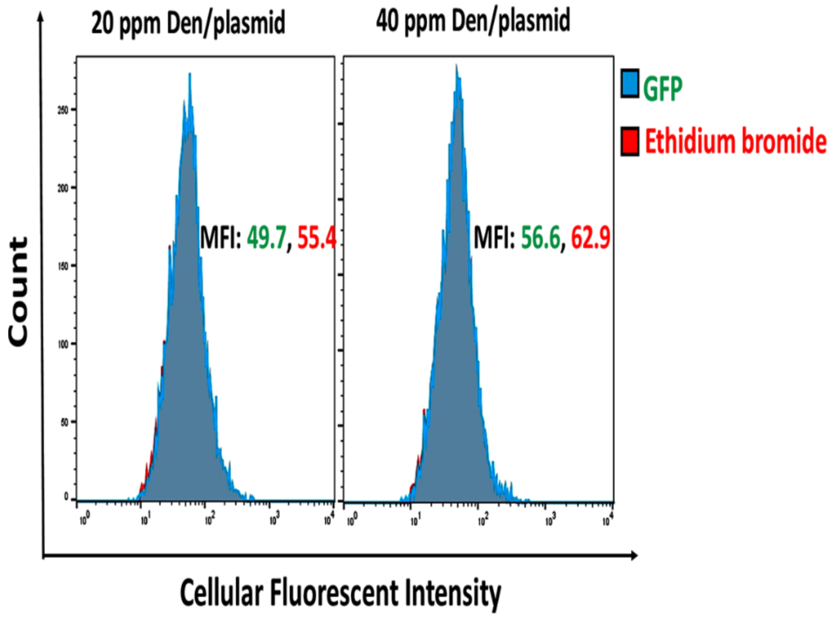

Figure S2. Quantitative internalization and expression of two different concentration of EtBr-labeled polyplexes by U87 cells. Results show that almost all of entered plasmids expressed after 48 h incubation in U87 cells. Blue: green fluorescent protein, Red: ethidium bromide
